# Solar Radiation as an Isolated Environmental Factor in an Experimental Mesocosm Approach for Studying Photosynthetic Acclimation of *Macrocystis pyrifera* (Ochrophyta)

**DOI:** 10.3389/fpls.2021.622150

**Published:** 2021-07-02

**Authors:** Paula S. M. Celis-Plá, José Luis Kappes, Félix L. Figueroa, Sandra V. Pereda, Karina Villegas, Robinson Altamirano, María Carmen Hernández-González, Alejandro H. Buschmann

**Affiliations:** ^1^Laboratory of Aquatic Environmental Research (LACER), Centro de Estudios Avanzados, Universidad de Playa Ancha, Viña del Mar, Chile; ^2^HUB Ambiental Universidad de Playa Ancha (UPLA), Vicerrectoría de Investigación Postgrado e Innovación, Universidad de Playa Ancha, Valparaíso, Chile; ^3^Centro de Investigación y Desarrollo de Ambientes y Recursos Costeros (Centro i-mar) and Centro de Biotecnología y Bioingenería (CeBiB), Universidad de Los Lagos, Puerto Montt, Chile; ^4^Department of Ecology and Geology, Faculty of Sciences, Institute of Biotechnology and Blue Development (IBYDA), University of Malaga, Malaga, Spain

**Keywords:** *Macrocystis pyrifera*, *in vivo* chlorophyll *a* fluorescence, phenolic compounds, photosynthetic pigments, dayle cycle experiments, mesocosm approach, brown algae

## Abstract

Solar radiation effects on the ecophysiology and biochemical responses of the brown macroalga *Macrocystis pyrifera* (L.) C. Agardh were evaluated using a mesocosm approach in Southern Chile. Treatments with different radiation attenuations were simulated with three vertical attenuation coefficients: (1) total (Kd = 0.8 m^−1^), (2) attenuated (Kd = 1.2 m^−1^), and (3) low (Kd = 1.6 m^−1^) radiation levels. Nutrient concentration and temperature did not show differences under the three light conditions. Photosynthetic activity was estimated by *in vivo* chlorophyll *a* (Chl*a*) fluorescence under the three light treatments as an isolated physical factor in both *in situ* solar radiation in the field. This was achieved using a pulse amplitude-modulated (PAM) fluorometera—Diving PAM (*in situ*). Photosynthetic activity and biochemical composition were measured in winter during two daily cycles (1DC and 2DC) in different parts of the thalli of the plant: (1) canopy zone, (2) middle zone, and (3) down zone, associated with different depths in the mesocosm system. Nevertheless, the *in situ* electron transport rate (ETR_*in situ*_) was higher in the exposed thalli of the canopy zone, independent of the light treatment conditions. The concentration of phenolic compounds (PC) increases in the down zone in the first daily cycle, and it was higher in the middle zone in the second daily cycle. The Chl*a* increased in the morning time under total and attenuated radiation in the first daily cycle. Solar radiation increasing at midday prompted the photoinhibition of photosynthesis in the canopy zone but also an increase in productivity and phenol content. Therefore, light attenuation in the water column drove key differences in the photo-physiological responses of *M. pyrifera*, with the highest productivity occurring in thalli positioned in the canopy zone when exposed to solar irradiance.

## Highlights

- Mesocosm experiments are a valuable tool for manipulating environmental factors in *Macrocystis pyrifera* through the photosynthetic activity in the thalli
- *Macrocystis pyrifera* shows high photoprotection and antioxidant capacity upon higher irradiance
- Photoacclimation in *M. pyrifera* is also based on the regulation of pigment composition
- The productivity in *M. pyrifera* decreased along the thalli

## Introduction

Habitat-forming, aquatic algal species, like kelps, can be affected at the individual or population level by variable light conditions within the water column, and they can present different patterns of photoacclimation, temperature, and nutrients, among others. In addition, their photosynthetic activity and growth often depend on other local environmental variables in the water column, such as nutrient content, temperature, and hydrodynamics (e.g., Wernberg et al., 2016; Fernández et al., 2020). Large kelps, such as *Macrocystis pyrifera* (L.) C. Agardh, can produce a floating canopy, and they are exposed to an array of predictable and unpredictable daily and seasonal changes. These include the levels of photosynthetically active radiation (PAR) and ultraviolet radiation (UVR) at different depths in the water column (Palacios et al., 2021). Too much or too little solar radiation can provoke negative impacts in kelp species that play a fundamental role in the structuring of coastal communities, thereby generating a subsequent loss of suitable habitat for other aquatic organisms (e.g., Graham, 2004; Olabarría et al., 2012). Seeking to better understand how solar radiation affects macroalgal growth and physiological parameters is a complex undertaking, one that requires the careful manipulation of different environmental factors (Celis-Plá et al., 2015).

The sporophytes of *M. pyrifera* are exposed to a wide range of daily and seasonal changes in PAR and UVR, with the latter known to harm biological organisms when in excess (Gómez and Huovinen, 2011). However, the macroalgae harbor differing species-specific sensitivity to irradiance, and this depends upon their morphology, position on the shore (intertidal/subtidal), as well as the life cycle stage (Bischof et al., 2006; Gómez and Huovinen, 2011), on the physiological acclimation capacities, adaptive properties of ecotypes/genotypes, and possible synergies with other environmental changes, among others (Varela et al., 2018). Macroalgae can survive and grow under full radiation by employing active photoprotection mechanisms, such as the accumulation of UV-screening substances, increasing antioxidant capacity, or high non-photochemical quenching (Hanelt and Figueroa, 2012; Figueroa et al., 2014; Celis-Plá et al., 2016). Thus, photoprotective compounds, such as some carotenoids and polyphenols, enable algae to cope with high radiation levels (Stengel et al., 2011) and dissipate the light energy of incident UV radiation (Goss and Jakob, 2010; Hanelt and Figueroa, 2012). Indeed, beyond certain threshold levels, high radiation can inhibit many biological processes (Barber and Andersson, 1992), potentially inducing the peroxidation of lipids and damaging nucleic acids (Wiencke et al., 2000). In this environmental complexity, the sporophyte populations themselves can modify water currents and sedimentation, and, in turn, light gradients, rendering interpretation of their physiological responses difficult.

In kelp species growing in temperate and cold waters, photosynthetic performance is often affected by the exposure of the plant to excess solar radiation (Buschmann et al., 2014a,b; Gómez et al., 2016; Häder, 2018). In this context, macroalgae are considered vulnerable, for instance, under high UV because the balance between mechanisms of photodamage, photoprotection, and photorepair is not in dynamic equilibrium (Murata et al., 2007; Celis-Plá et al., 2016). Moreover, excess radiation can disrupt the electron transport chains in chloroplasts by inducing an energy transfer to oxygen, overproduction of reactive oxygen species (ROS), and subsequent oxidative stress and damage (Heinrich et al., 2012). To increase their ability to assimilate the sunlight and transform it into chemical energy, macroalgae have evolved key morphological adaptations. *M. pyrifera* sporophytes use positive buoyancy to intercept solar radiation at the sea surface (Buschmann et al., 2006; Schiel and Foster, 2015) and have the capacity to modify their pigment content by changing the number and/or size of the light-harvesting antennae (Buschmann et al., 2014a; Hurd et al., 2014). Nevertheless, in these situations of high or limiting light availability, the multiple interactions with nutrient availability, temperature, and CO_2_ concentrations could produce synergistic or antagonistic interactions, which are difficult to disentangle and understand under field conditions (Buschmann et al., 2014a; Celis-Plá et al., 2015, 2017).

Experimental protocols with controlled or semi-controlled conditions in mesocosms can be used to study and learn how macroalgae physiologically manage the complex environmental conditions they must face in nature; also, it has been demonstrated that physiological capacities (like photosynthesis) in blade portions can be lower than in blades attached to the sporophyte. But because large-sized macroalgae with complex morphology, such as *M. pyrifera*, can modify currents (Gaylord et al., 2003) that, in turn, alter diffusion of nutrients and gases (Hurd, 2017); several authors have called for avoiding unrealistic laboratory experimental designs using tissue portions normally extracted from blades (Figueroa and Korbee, 2010; Drobnitch et al., 2017). For these reasons, other researchers have studied growth responses, photosynthesis, in outdoor tanks conditions (Cabello-Pasini et al., 2000; Figueroa et al., 2021), in laboratory conditions (Colombo-Pallotta et al., 2006), and/or nutrient uptake under field conditions (Edwards and Kim, 2010; Celis-Plá et al., 2015; Palacios et al., 2021).

Short-term (days) mesocosm studies give information on rapid acclimation to environmental conditions as climate change factors (Stengel et al., 2014; Figueroa et al., 2019, 2021), whereas long-term (months, years) mesocosm studies give information on adaptation to the variations in the environmental condition in a scale that allows reaching relevant conclusion of the vulnerability and adaptation to a climate change factor under different scenarios (Liborissen et al., 2005; Harley et al., 2012; Sordo et al., 2016). Also, transplant experiments from cold-nutrient rich to warmer-nutrient poor concentrations performed during the 1980's expanded our understanding of how large, morphologically complex kelps (*M. pyrifera*) can respond to light under those environmental factors (e.g., Gerard, 1982, 1984) but cannot decouple the environmental factors that change between the transplant sites. More specifically, concerning light harvesting, photosynthetic, and productivity potential of large kelps, most of the recent work has been carried out by measuring photosynthetic performance on tissues obtained at different water depths and acclimated to different light conditions (Palacios et al., 2021). However, when trying to understand these large kelps, it is not possible to rely on integrating independent measurements of individual sporophyte performance in growth and productivity. Because these organisms may modify the environment due to their size, parts of their thalli may be affected by different light, temperature, salinity, and nutrient conditions, and these organisms can translocate metabolites along their thalli structures (Bartsch et al., 2008). Our outdoor mesocosm system offers an alternative approach to study and separate the vertical effects of light along the water column, temperature, and nutrients on a whole, large kelp sporophyte (Buschmann et al., 2014a). Nevertheless, manipulating the mesocosm system requires extremely strict protocols to avoid artifacts that may confound the results under such experimental conditions (Celis-Plá et al., 2017).

The current study explored the effects of light attenuation on *M. pyrifera* sporophytes along an artificial depth gradient in a mesocosm system in three treatments characterized by light attenuation coefficients: total (Kd = 0.8 m^−1^), attenuated (Kd = 1.2 m^−1^), and low (Kd = 1.6 m^−1^) levels of radiation. We estimated the photosynthetic performance of *M. pyrifera* through *in vivo* chlorophyll *a* fluorescence and measured biochemical responses at different thalli levels to evaluate if light attenuation affects the physiological responses in a differential way at the canopy, middle, and down zones of the kelp thalli. Photosynthetic activity was determined in three parts of the kelp thalli, according to their depth within the water column: canopy, middle, and down zones. Applying this experimental protocol enhanced our understanding of how daily light attenuation along with a depth gradient influences photosynthetic production and affects key biochemical compounds in *M. pyrifera* (Buschmann et al., 2014b; Camus et al., 2018; Varela et al., 2018; Palacios et al., 2021).

## Materials and Methods

### Sampling

*Macrocystis pyrifera* (Laminariaceae: Laminariales) adult sporophytes (ca. 2.5 m in length) were collected at 2- to 4-m depth by scuba diving in Carelmapu, Chile (41°44′ S, 73°44′ W) during winter (July 2017). To avoid damaging these alga samples, they were transported in coolers to the mesocosm system located at the Centro i-mar (Universidad de Los Lagos in Puerto Montt).

### Experimental Design and Abiotic Parameters

**Figure 1:**
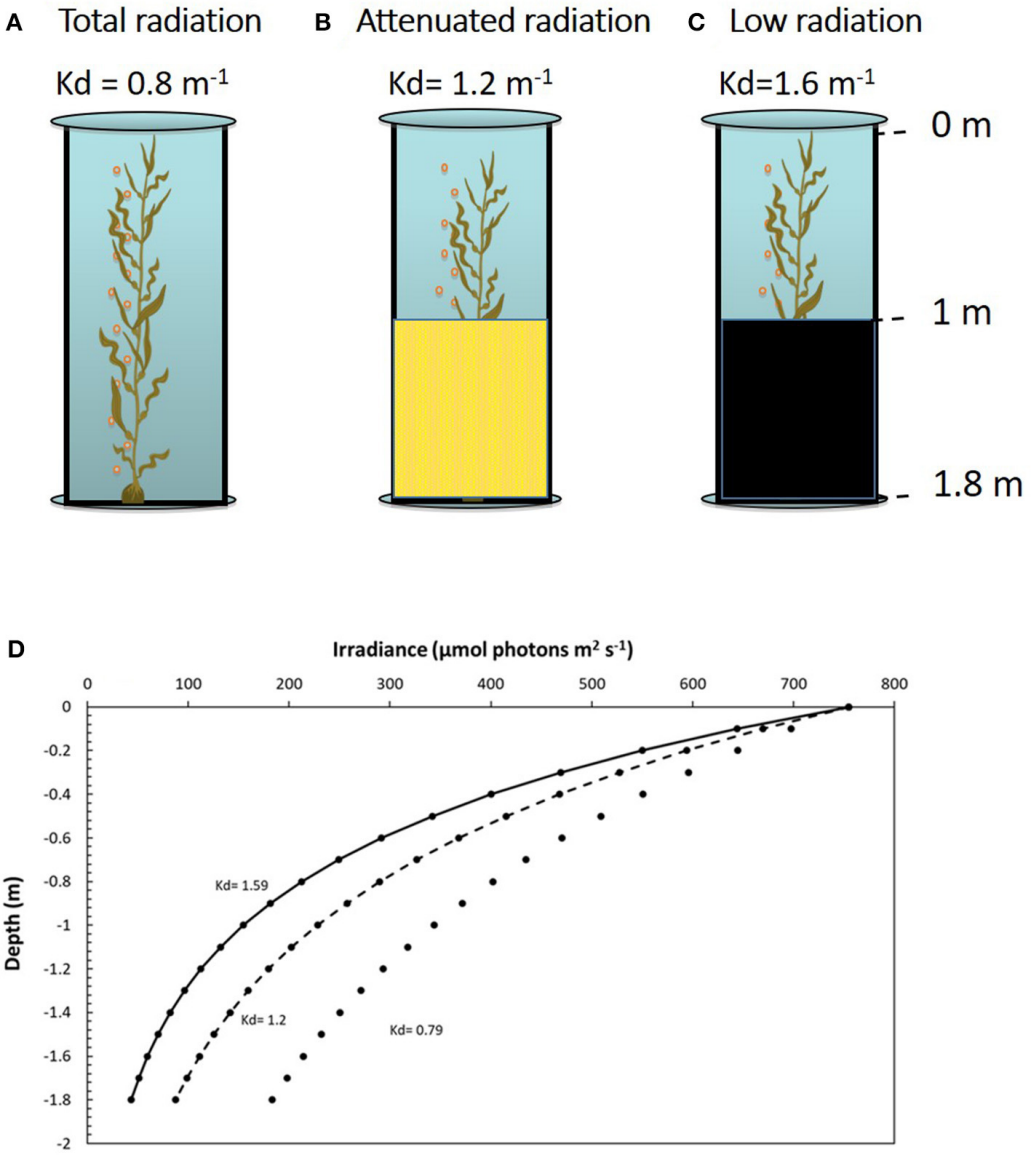
Mesocosm system with adult sporophytes of *Macrocystis pyrifera* inside the tanks. Shown are the three treatments of **(A)** total radiation (no filter used), **(B)** attenuated radiation (yellow mesh covering the bottom half), and **(C)** low radiation (asphalt felt covering the bottom half). **(D)** Photosynthetically active radiation (PAR) in the water column according to the Kd in each treatment; total radiation, Kd = 0.8 m^−1^; attenuated radiation, Kd = 1.2 m^−1^; low radiation, Kd = 1.6 m^−1^.

The sporophytes of *M. pyrifera* were installed in a 12-tank outdoor mesocosm for acclimation to the experimental conditions (7 days). The outdoor mesocosm system consisted of 400-L, semitransparent fiberglass cylindric tanks, 1.8 m in height and 0.6 m in diameter (Figures 1A–C). Three tanks remained without kelp to allow for water quality control, and all the treatments and replicated tanks (*n* = 3) were distributed randomly to avoid position biases (Hurlbert, 1984). Each tank contained three or four fertile sporophytes (2.5-m long) to avoid pseudo-replication in time (see Supplementary Tables S1, S2). Tanks were continuously aerated using an air blower, and the seawater was completely replaced every 3 days by pumping seawater from a depth >3 m at the coastline at Centro i-mar (41°49′ S, 72°98′ W), allowing to maintain homogeneous temperature, nutrients, pH, along with the water column (Buschmann et al., 2014a). In this experiment, nutrients were not added to the experimental unit, simply because, in winter, the nitrate level in the water was always well over 15 μM (Camus et al., 2018).

Two daily cycle experiments were conducted in the mesocosm, henceforth 1DC (first daily cycle) and 2DC (second daily circle). Three solar irradiance treatments corresponding to different light attenuation coefficients (Kd) were randomly assigned to the mesocosm tanks: *total radiation* (Figure 1A, 100% solar radiation where light penetration had a Kd = 0.8 m^−1^); *attenuated radiation* (Figure 1B, with mesh covering the bottom half to generate a light penetration of Kd = 1.2 m^−1^), and *low radiation* (Figure 1C, with dense mesh around the bottom half, producing a light penetration of Kd = 1.6 m^−1^). Measurements for ecophysiological responses were made in three structural zones of the sporophytes: (1) canopy zone; (2) middle zone, and (3) down zone. These measurements were carried out during the hours of daylight each day at 8:00, 13:00, and 17:00 h.

Irradiance of PAR (λ = 400–700 nm; expressed in μmol photons m^−2^ s^−1^) and UVA (315–400 nm; in Wm^−2^) were continuously recorded every 5 min during the experimental period by QSO-SUN 2.5V and USB-SU 100 sensors, respectively. Each sensor and its respective data logger (HOBO U12-006, Onset Computer Corporation, Cape Cod, MA, USA) were placed inside a polycarbonate box (Otter Box 3000) at 1.8-m depth in each tank. For each treatment, the diffuse downward attenuation coefficient (Kd) of PAR was calculated, following Quintano et al. (2017). The Kd was determined for each experimental period by applying the Beer–Lambert law equation:

(1) $$\begin{array}{r} E_{d}\left(z\right)=E_{d}\left(0\right)\;\cdot e^{-Kd.z} \end{array}$$

where *E*_*d*_(*z*) is the irradiance (PAR) measurement at depth *z* in each tank, *E*_*d*_(0) is the irradiance measurement at the underwater surface of the sensor, Kd is the PAR-diffuse attenuation coefficient, and *z* is depth. Due to the light, the field was heterogeneous, by using meshes out of the tanks from 1- to 1.8-m depth (see Figure 1), Kd was calculated into water mass from (1) a surface to 1 m and (2) 1 m to 1.8 m. Table 1 shows the Kd data at three different times during the day (8:00, 13:00, and 17:00 h). In addition, the depth of 50, 15, 10, and 1% of surface radiation was also calculated. The water temperature was continuously measured by a HOBO U22 water temp/light UA-002-64 logger (Onset Computer Corporation).

**Table 1:** Kd data in the three different treatments: total, attenuated, and low radiation, for the mesocosm system, and the surface radiation to the different depths at 50, 15, 10, and 1%.

**Variables**	**Hours**	**Total radiation**	**Attenuated radiation**	**Low radiation**
Kd1 (0–1 m)	8:00	0.802	1.200	1.586
	13:00	0.780	1.196	1.610
	17:00	0.792	1.192	1.598
Average Kd1		0.791	1.196	1.593
Kd2 (1–1.8 m)	8:00	0.783	1.238	1.627
	13:00	0.819	1.243	1.625
	17:00	0.804	1.207	1.591
Average Kd2		0.801	1.229	1.614
Average 0–1.8 m		0.796	1.215	1.603
D 50%		0.871	0.570	0.432
D 25%		1.742	1.141	0.865
D 10%		2.893	1.895	1.436
D 1%		8.325	5.454	4.134

### Photosynthesis as *in vivo* Chlorophyll a Fluorescence

#### *In situ* Measurements

After the acclimation period for the kelp, the *in situ* measurements were performed in the mesocosm system in terms of the *in vivo* chlorophyll *a* fluorescence of the Photosystem II (PSII). These measurements were quantified using a portable pulse amplitude-modulated (PAM) diving fluorometer (Walz GmbH, Effeltrich, Germany), as described in Celis-Plá et al. (2016). Photosynthetic activity was quantified in the blades along the thalli in the canopy, middle, and down zones at 8:00, 13:00, and 17:00 h during the 1DC and 2DC after 7 days of acclimation. Data from previous tests allowed us to determine the number of measurements to be collected on each blade, taking into consideration apical, meristematic, marginal, and central sections. In recognition of the variability observed, six measurements per blade were taken.

The effective quantum yield (Y_II_ or Δ*F/F*_*m*_′) was calculated according to Schreiber et al. (1995):

(2) $$\begin{array}{r} Y_{II}=\left(F_{m}-F\right)/F_{m^{\prime }} \end{array}$$

where *F*${}_{m^{\prime }}$ is the maximal fluorescence induced with a saturating blue-light pulse and *F* is the current steady-state fluorescence in light-adapted algae. The *in situ* electron transport rate (ETR_*in situ*_) through PSII was calculated from

(3) $$\begin{array}{r} ETR_{in\;situ}=Y_{II}\times E_{PAR}\times A\times F_{II} \end{array}$$

where E_PAR_ is the incident PAR irradiance when a measurement is made, A is the thallus absorptance value—the fraction of incident irradiance absorbed by the algae according to Celis-Plá et al. (2014)—and *F*_*II*_ is the fraction of chlorophyll *a* associated with PSII, with the value in brown algae set to 0.8, according to Grzymski et al. (1997) and Figueroa et al. (2003).

### Biochemical Analyses

Tissue samples were stored at −80°C before the analyses of pigment contents, phenolic compounds, and antioxidant activity.

Pigment contents as chlorophyll *a* (Chl*a*), chlorophyll_*c*1+*c*2_, (Chl_*c*1+*c*2_), and fucoxanthin (Fux) were extracted from 20-mg FW of blades (FW) according to Seely et al. (1972). After 5 min, the optical density or absorbance was determined spectrophotometrically (Model Infinite M 200 Pro, TECAN, Hombrechtikon, Switzerland) (Celis-Plá et al., 2018). Pigment concentrations were expressed as mg g^−1^DW after determination of the FW:DW ratio.

(4) $$\begin{array}{rc} \text{Chl}a & =\left({\text{A}}_{665}\right)/72.5 \end{array}$$

(5) $${\text{Chl}}_{cl+c2}={\text{(A}}_{\text{631}}+{\text{A}}_{\text{582}}-\text{0}{\text{.297A}}_{\text{665}}\text{)}/\text{61.8}$$

(6) $$\begin{array}{l} Fux=({\text{A}}_{480}-0.722({\text{A}}_{631}+{\text{A}}_{582}-0.297{\text{A}}_{665})\\ \;-0.049{\text{A}}_{665})/130 \end{array}$$

Total phenolic compounds (PC) were determined using 25-mg FW of blades, first pulverized with a mortar and a pestle, containing sea sand and 2.5 ml of 80% methanol. After storing the samples overnight at 4°C, the mixture was centrifuged at 2,253 *g* for 30 min at 4°C, and the ensuing supernatant was collected. Total PC was determined colorimetrically, using the Folin-Ciocalteu reagent (Folin and Ciocalteu, 1927), for which phloroglucinol (1.3.5-trihydroxybenzene, Sigma P-3502) served as the standard and the sample absorbance determined at 760 nm (Celis-Plá et al., 2016). The PC content was expressed as mg g^−1^ DW, and the results are expressed as the mean ± one SE, with these compounds determined spectrophotometrically (Model Infinite M 200 Pro, TECAN, Hombrechtikon, Switzerland).

Antioxidant capacity (AA%) was determined using the DPPH (2.2-diphenyl-1-picrylhydrazyil) method applied to the same extract as used for the above PC analysis (1.27 mM of DPPH is used in 80% methanol) (Celis-Plá et al., 2016). The antioxidant activity was measured in 150 μl of algae extract and 150 μl of DPPH immediately and then again after 30 min at room temperature (~20°C). The reaction was determined at 517 nm, and then the antioxidant capacity of a given sample was calculated as

(7) $$\begin{array}{r} \text{AA}\%\;=\;\left[\left(\text{A}\left(\text{initial}\right)-\text{A}\left(\text{3}0\text{min}\right)\right)\;/\text{A}\left(\text{initial}\right)\right]\times \text{1}00 \end{array}$$

Internal nitrogen (N) and carbon (C) contents were determined in fronds, using an element analyzer CNHS-932 model (LECO Corporation, St. Joseph, MI, USA). Nitrogen and carbon were expressed as mg g^−1^ dry weight (DW) after determining the fresh weight (FW) to DW ratio in the tissue of *M. pyrifera*.

### Statistical Analyses

Physiological and biochemical responses were analyzed using ANOVAs, including three fixed factors: (i) radiation treatments (three levels: total, attenuated, and low radiation), (ii) time (three levels: 8:00, 13:00, and 17:00 h), and (iii) zones of thalli (three levels: canopy, middle, and down zones) (*n* = 3, mean ± SE; Supplementary Table S3). Homogeneity and homoscedasticity of variance were assessed using Cochran tests and by visual inspection of the residuals, and the Student Newman Keuls (SNK) test was performed after significant ANOVA interactions (Underwood, 1997). All data conformed to the homogeneity of variance assumption. Analyses were carried out in SPSS v.21 software (IBM, Armonk, NY, USA). Finally, Pearson correlation coefficients were calculated and tested between physiological variables, namely, ETR _*insitu*_, as well as biochemical variables—nitrogen and carbon content, Chl*a*, Chl_*c*1+*c*2_, *Fux*, PC, and AA%—for both 1DC and 2DC (Sigmaplot v.14, Systat Software, San Jose, CA, USA).

## Results

### Physical–Chemical and Photosynthetic Activity

Average Kd values were lower under total radiation treatment (K_d, 0−1m_ = 0.791 and K_d, 1−1.8m_ = 0.801) than under attenuated radiation (K_d, 0−1m_ = 1,196 and K_d, 1−1.8m_ = 1,229), followed by low radiation (K_d, 0−1m_ = 1,593 and K_d, 1−1.8m_ = 16,141). The Kd values in the bottom part of the tanks (1–1.8-m depth) were slightly higher than in surface parts (0–1-m depth). Slight variations in Kd during the daily cycle were observed. The average Kd of the two measurements, i.e., 0–1 m and 1–1.8 m was 0.8 m^−1^, 1.2 m^−1^, and 1.6 m^−1^ for the total, attenuated, and low radiation treatments, respectively (Table 1, Figure 1D). The differences in the transparency of the water column can be also illustrated by the different depths in which 50 to 0.1% of surface irradiance was reached. In the total radiation tanks, 25% of surface irradiance was reached at 1.742 m (close to the bottom of the tank, i.e., 1.8 m), whereas, in attenuated tanks, was only 1.14 m followed by low-irradiance tanks 0.865 m. The 10% of surface irradiance could reach 2.89 m in total radiation (a higher depth than the used tanks), whereas, under attenuated, light conditions reach the bottom of the tanks (1.89 m) but only 1.4 m under low radiation tanks. Finally, 1% of surface radiation could reach 8.325, 5.454, and 5.4.134 m in total, attenuated and low radiation tanks, respectively.

UVA irradiance in surface water ranged from 3 to 3.5 Wm^−2^ at around noon in the first daily cycle (1DC), whereas, in the second daily cycle (2DC), it was between 3.5 and 4 Wm^−2^ at 13:00 h (data not shown). Average seawater temperature in the 1DC experiment had a minimal value of 8.50 ± 0.01°C in the morning (low-radiation treatment), reaching its maximal value of 13.45 ± 0.1°C at 17:00 h in the total radiation treatment (Supplementary Table S1). In 2DC, the temperature had a minimal value at 8:00 h of 5.64 ± 0.05°C and a maximal value of 10.94 ± 0.13°C at 17:00 h; both values registered in the total radiation treatment (mean ± SE, *n* = 12) (Supplementary Table S1). Nitrate content was measured along the water column of each tank; no significant differences were found among the different light attenuation treatments (Supplementary Table S2).

**Figure 2:**
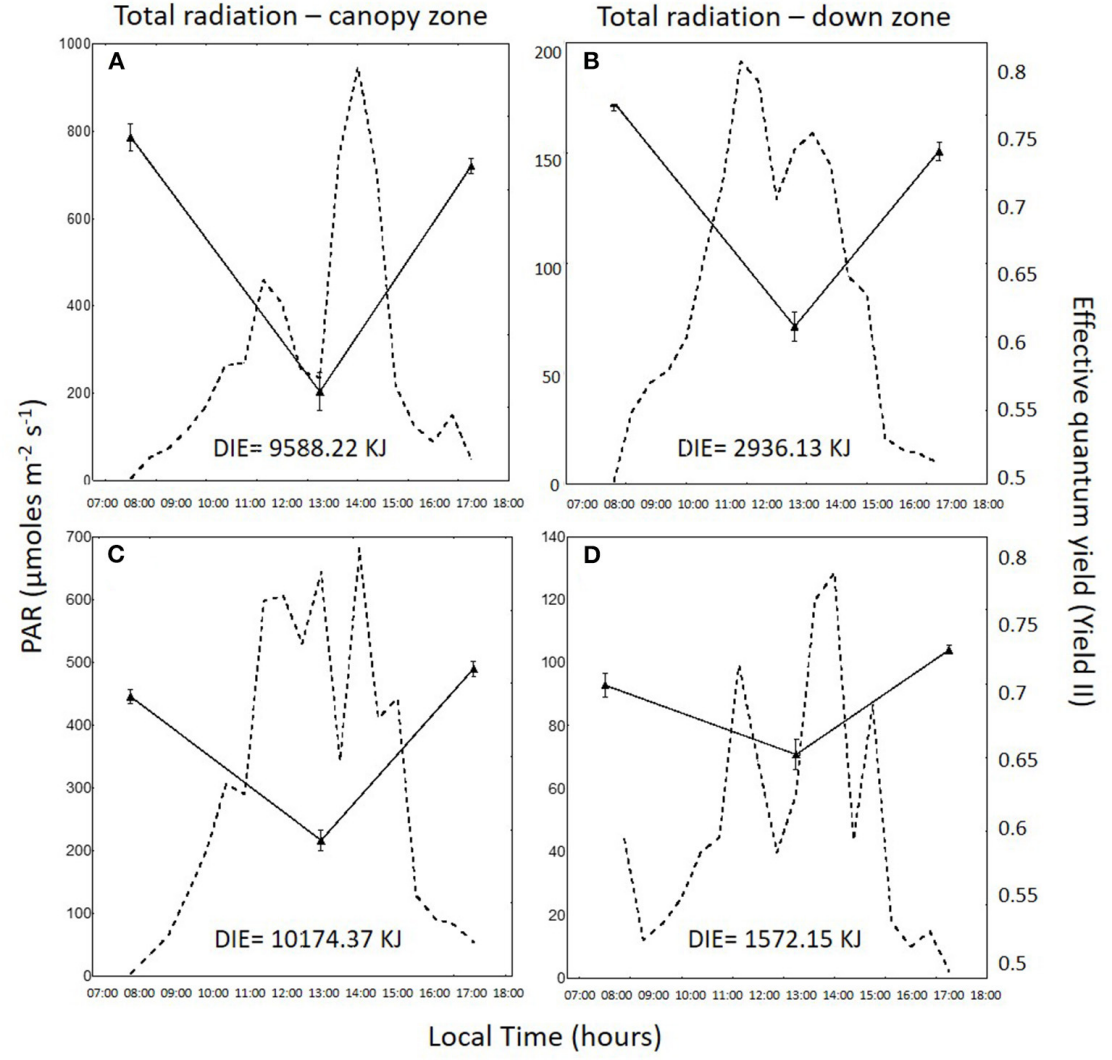
Photosynthetically active radiation (PAR: 400–700 nm, in μmol photons m^−2^ s^−1^, dotted lines) and the effective quantum yield (Y_II_) in *Macrocystis pyrifera* fronds (continuous lines) in the total radiation treatment during the first daily cycle under the canopy zone **(A)** and the down zone **(B)**; likewise, in the second daily cycle under the canopy zone **(C)** and the down zone **(D)**.

**Figure 3:**
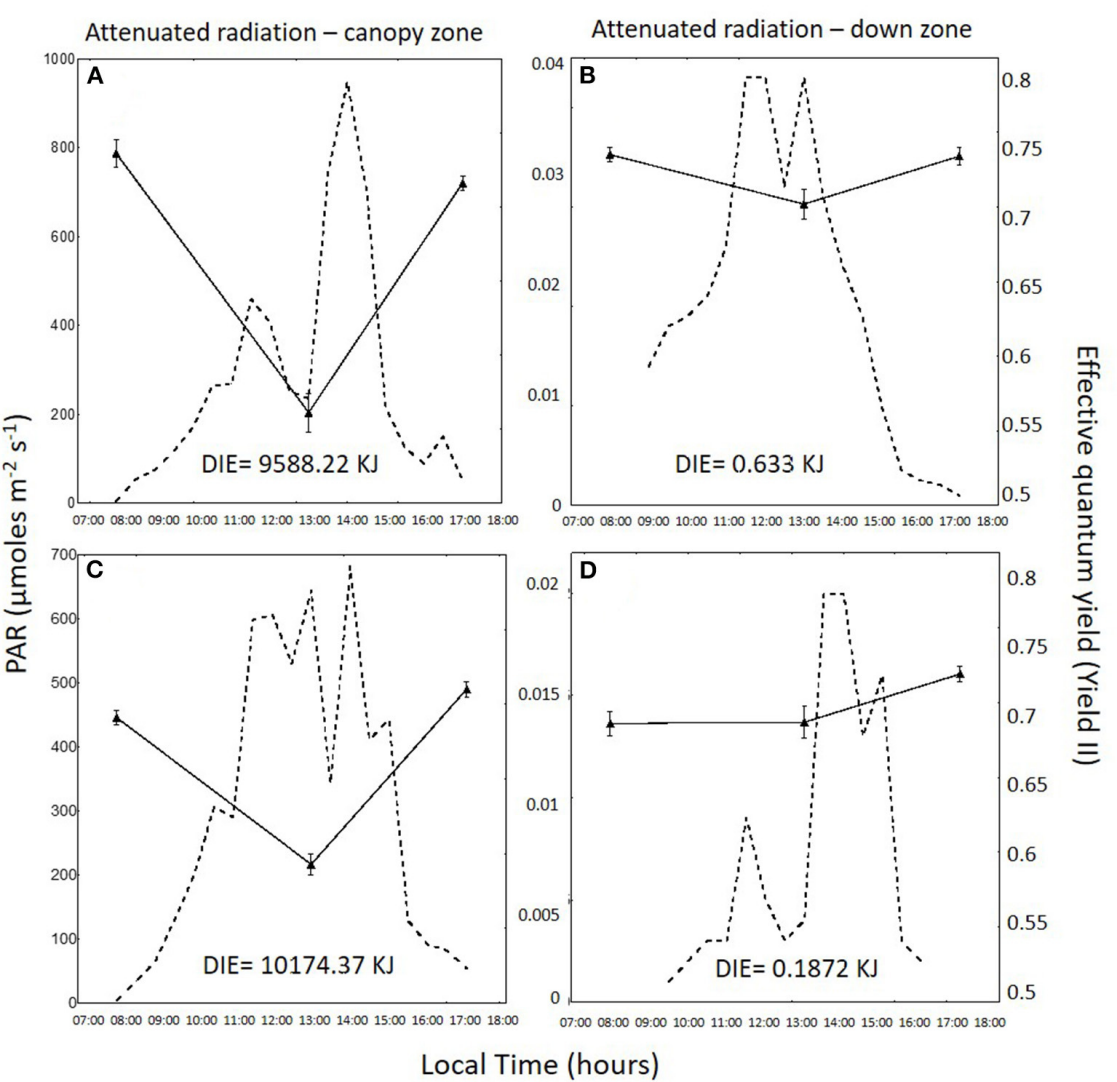
Photosynthetically active radiation (PAR: 400–700 nm, in μmol photons m^−2^ s^−1^, dotted lines) and the effective quantum yield (*Y*_*II*_) in *Macrocystis pyrifera* fronds (continuous lines) in the attenuated radiation treatment during the first daily cycle under the canopy zone **(A)** and the down zone **(B)**; likewise, in the second daily cycle under the canopy zone **(C)** and the down zone **(D)**.

**Figure 4:**
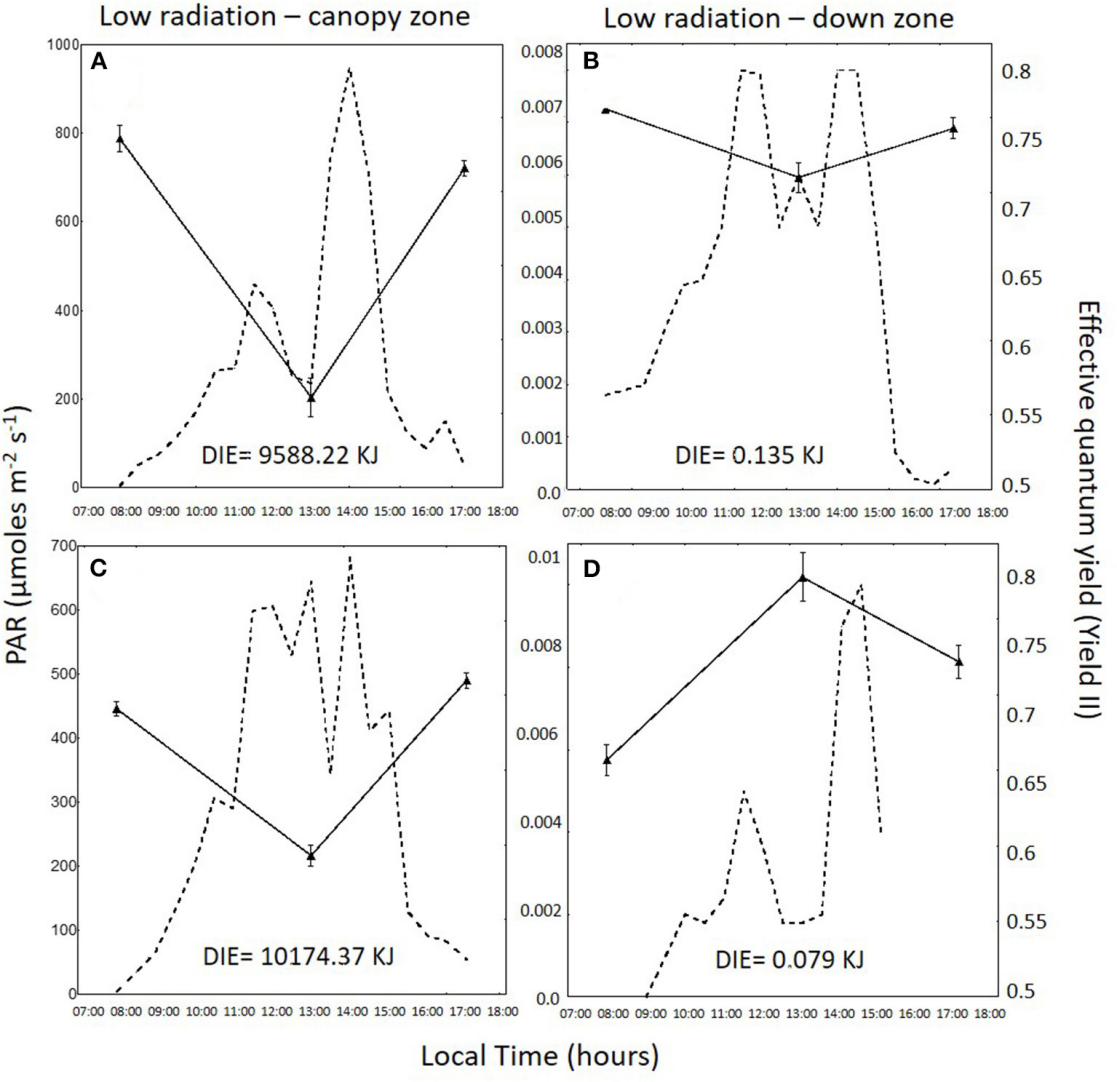
Photosynthetically active radiation (PAR: 400–700 nm, in μmol photons m^−2^ s^−1^, dotted lines) and the effective quantum yield (*Y*_*II*_) in *Macrocystis pyrifera* fronds (continuous lines) in the low-radiation treatment during the first daily cycle under the canopy zone **(A)** and the down zone **(B)**; likewise, in the second daily cycle under the canopy zone **(C)** and the down zone **(D)**.

Daily variation in irradiance and effective quantum yield in the total radiation treatment for the canopy zone, the middle zone, and the down zone are shown for both daily cycles in Figures 2–**4**. In 1DC, the daily integrated irradiance was three times higher in the canopy than the down zone for the exposed fronds, whereas, in the 2DC, it was about 6.5 times higher (Figure 2). The decrease in *Y*_*II*_ at 13:00 was greater in the canopy zone when compared with the down zone exposure at the bottom of the tanks. The decline in *Y*_*II*_ in the canopy zone under the attenuated light treatment (Figure 3) followed the same pattern as that under total radiation (Figure 2); however, due to the higher attenuation of the light, the decrease of irradiance in the bottom was higher compared with the total radiation treatment. The daily integrated irradiance (DIE) was higher at the top than at the bottom of the tank. Thus, in the DC1, the DIE in the canopy zone was 3.26 higher than in the down zone, whereas, in the DC2, was 6.47 (Figure 2). The differences in the attenuated and low radiation tanks were much higher (Figures 3, 4). In the attenuated radiation treatment, Y_II_ presented a slight decrease or no decrease at 13:00 (Figure 3). In the low-radiation treatment (Figure 4), with its higher Kd, the reduction in doses in the down zone still exceeded those observed in the attenuated radiation treatment. In the down zone, Y_II_ underwent a slight decrease (the first daily cycle) and an increase in the 2DC at ca.13:00 (Figure 4).

**Figure 5:**
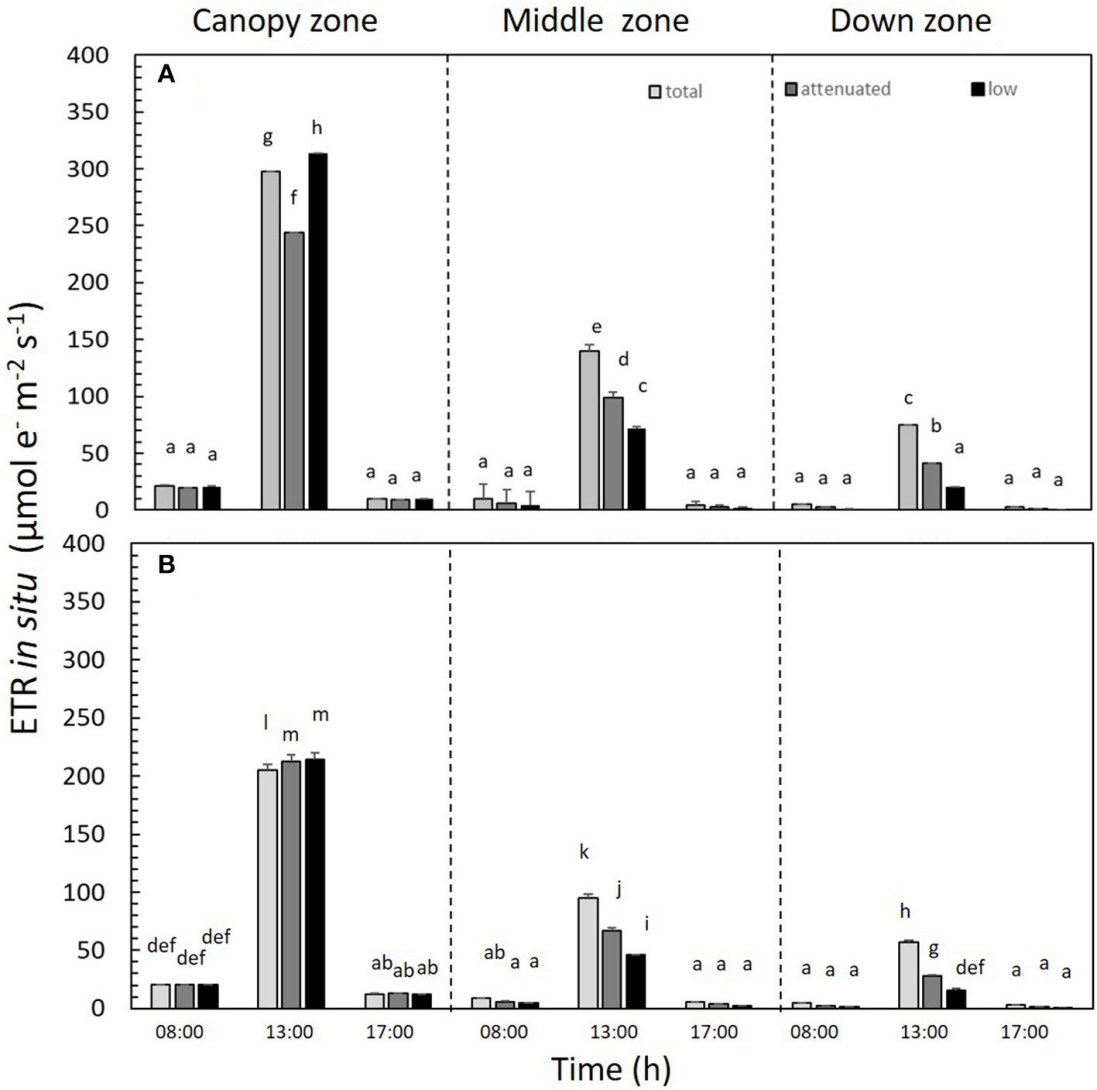
*In situ* electron transport rate (ETR_*in situ*_) in *Macrocystis pyrifera* fronds in the **(A)** first daily cycle experiment and **(B)** second daily cycle experiment under different solar irradiance treatments: total, attenuated, and low radiation at 8:00, 13:00, and 17:00 h. The ETR_*in situ*_ was measured in three zones of the alga: canopy, middle, and down (mean ± SE, *n* = 3). Lower-case letters denote significant differences after the SNK test.

ETR_*in situ*_ differed significantly among all treatments (*p* < 0.05). ETR_*in situ*_ was higher at 13:00 h in all treatments in the canopy zone. This productivity index decreased in both daily cycles at 8:00 and 17:00 h (Figures 5A,B and Supplementary Table S3). In both daily cycles, the ETR_*in situ*_ of the canopy zone under low radiation was higher compared with the other two treatments—total and attenuated. Thus, in the treatment with the highest light attenuation (i.e., low radiation), the canopy zone presented the highest ETR values (Supplementary Table S3).

### Biochemical Responses

**Figure 6:**
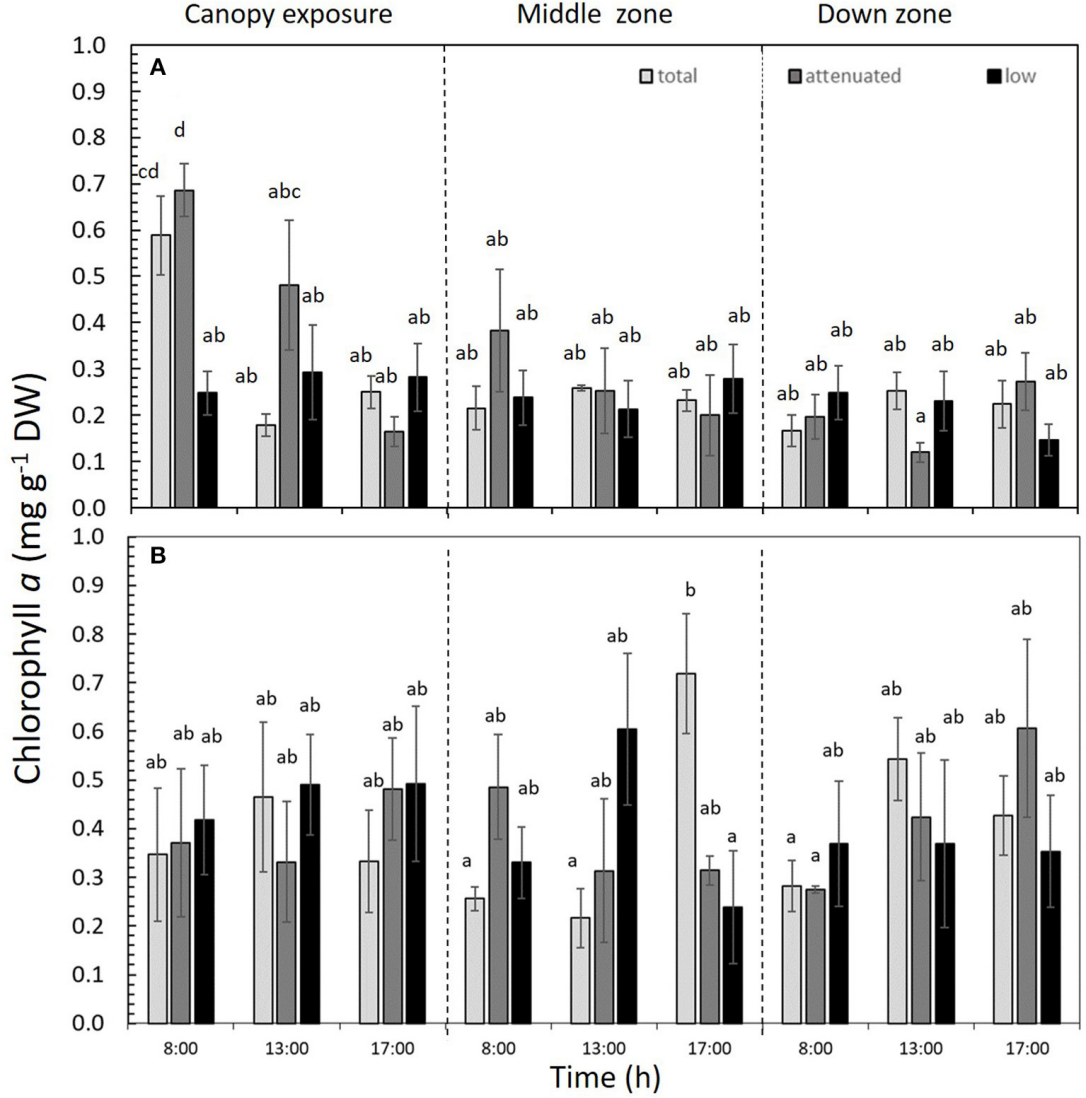
Chlorophyll *a* (Chl*a*) content in *Macrocystis pyrifera* fronds during the first daily cycle experiment **(A)** and the second daily cycle experiment **(B)** at three time points (8:00, 13:00, and 17:00 h) in the total, attenuated, and low radiation treatments. Chl*a* was measured in three zones of the alga: canopy, middle, and down (mean ± SE, *n* = 3). Lower-case letters denote significant differences after the SNK test.

Chl*a* was significantly affected by the interaction among all three factors (*p* < 0.05) in both 1DC and 2DC (Figure 6 and Supplementary Table S3). In 1DC, Chl*a* content was highest at 8:00 h in the canopy zone of *M. pyrifera* under both total and attenuated radiation treatments (Figure 6A and Supplementary Table S3). In 2DC, the Chl*a* increased significantly under total radiation at 17:00 h (Figure 6B and Supplementary Table S3). The Chl_*c*1+*c*2_ was higher in the attenuated and low radiation treatments at 8:00 and 13:00 h for 1DC, but it was higher in 2DC at 17:00 h under attenuated radiation (Table 2 and Supplementary Table S3). *Fux* was higher in 1DC at 8:00 h under total radiation, yet, in 2DC, it was higher irrespective of the time of day under the low-radiation treatment in the canopy zone (Table 2 and Supplementary Table S3).

**Table 2:** Chlorophyll _c1+c2_, fucoxanthin (*Chl*_*c*1+*c*2_ and *Fux*, expressed in mg g^−1^ DW) and antioxidant activity (AA%, in percent) during the 1DC and 2DC in the different solar irradiance treatments: total, attenuated, and low radiation at three times during the day.

			**Total radiation**			**Attenuated radiation**			**Low radiation**		
		**Time**	***Chl_***c*1+*c*2**_***	***Fux***	***AA%***	***Chl_***c*1+*c*2**_***	***Fux***	***AA%***	***Chl_***c*1+*c*2**_***	***Fux***	***AA%***
First daily cycle	Canopy zone	8:00	0.29 ± 0.3	0.12 ± 0.13	43.13 ± 18.15	0.38 ± 0.25	0.16 ± 0.11	51.91 ± 14.3	0.27 ± 0.28	0.12 ± 0.13	67.24 ± 13.18
		13:00	0.17 ± 0.05	0.07 ± 0.02	62.02 ± 10.5	0.17 ± 0.07	0.07 ± 0.02	58.94 ± 10.45	0.19 ± 0.12	0.08 ± 0.05	57.94 ± 4.18
		17:00	0.2 ± 0.09	0.09 ± 0.04	58.99 ± 8.22	0.21 ± 0.08	0.09 ± 0.03	55.84 ± 15.85	0.16 ± 0.07	0.06 ± 0.03	59.06 ± 16.53
	Down zone	8:00	0.21 ± 0.12	0.09 ± 0.05	63.94 ± 15.28	0.35 ± 0.29	0.16 ± 0.14	57.91 ± 14.09	0.32 ± 0.14	0.14 ± 0.06	63.36 ± 9.32
		13:00	0.2 ± 0.11	0.09 ± 0.04	59.17 ± 9.38	0.36 ± 0.43	0.16 ± 0.2	57.13 ± 10.3	0.25 ± 0.09	0.11 ± 0.04	61.03 ± 12.36
		17:00	0.16 ± 0.09	0.07 ± 0.04	63.62 ± 14.28	0.18 ± 0.09	0.08 ± 0.04	50.65 ± 10.79	0.18 ± 0.13	0.08 ± 0.06	69.92 ± 7.85
Second daily cycle	Canopy zone	8:00	0.24 ± 0.26	0.1 ± 0.12	31.92 ± 9.18	0.19 ± 0.16	0.08 ± 0.07	33.53 ± 10.02	0.3 ± 0.27	0.12 ± 0.12	42.01 ± 11.77
		13:00	0.24 ± 0.16	0.1 ± 0.07	33.72 ± 21.42	0.2 ± 0.16	0.08 ± 0.07	30.44 ± 12.88	0.3 ± 0.19	0.13 ± 0.08	43.57 ± 24.75
		17:00	0.25 ± 0.19	0.11 ± 0.09	42.34 ± 8.07	0.28 ± 0.12	0.12 ± 0.06	35.77 ± 11.42	0.26 ± 0.2	0.11 ± 0.09	40.36 ± 21.73
	Down zone	8:00	0.19 ± 0.22	0.08 ± 0.1	42.23 ± 6.05	0.28 ± 0.18	0.12 ± 0.08	26.98 ± 11.18	0.27 ± 0.13	0.11 ± 0.06	55.34 ± 13.41
		13:00	0.25 ± 0.16	0.1 ± 0.08	49.42 ± 24.04	0.19 ± 0.14	0.07 ± 0.06	31.84 ± 17.72	0.24 ± 0.19	0.1 ± 0.09	53.25 ± 16.68
		17:00	0.27 ± 0.23	0.11 ± 0.11	46.11 ± 15.45	0.36 ± 0.13	0.15 ± 0.06	33.11 ± 20.32	0.22 ± 0.11	0.09 ± 0.05	48.89 ± 11.42

*The pigments contents and AA% were determined in two different zones of the thalli (canopy and down zones) (mean ± SE, n = 3)*.

**Figure 7:**
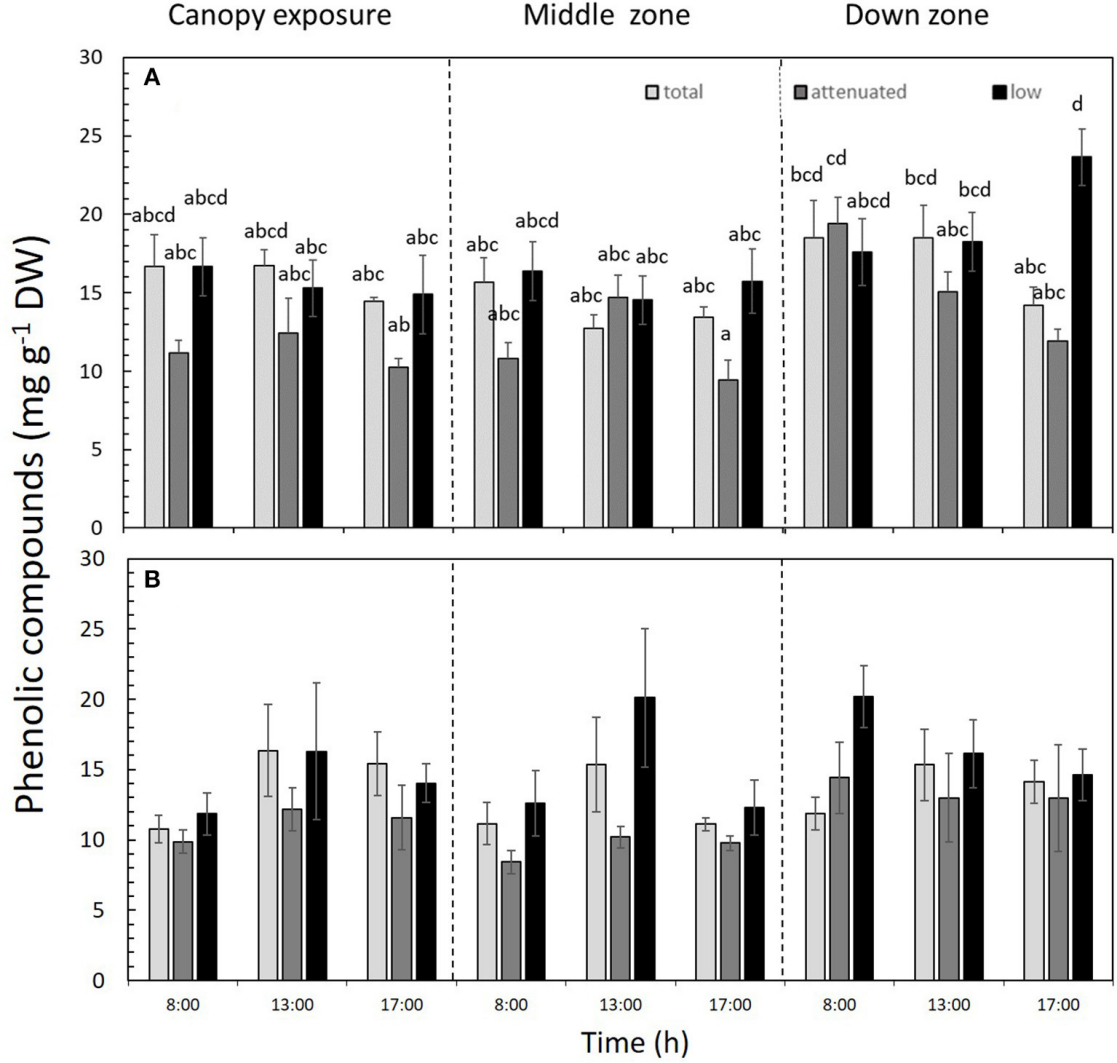
Phenolic compounds (PC, mg g^−1^ DW) in *Macrocystis pyrifera* fronds during the first daily cycle experiment **(A)** and the second daily cycle experiment **(B)** according to the different solar irradiance treatments (total, attenuated, and low radiation) and time points (8:00, 13:00, and 17:00) and zones of the macroalga (canopy, middle, and down). Shown is the mean ± SE (*n* = 3). Lower-case letters denote significant differences after the SNK test.

The PC contents were significantly affected by the interaction between the light attenuation treatments × time × zones of the alga (*p* < 0.05) in both daily cycle experiments (Figure 7). In 1DC, the PC was highest under low radiation in the down zone of the sporophyte at 17:00 h (Figure 7A and Supplementary Table S3), while, in 2DC, more PC occurred under total radiation *vis-à-vis* the attenuated and low-radiation treatments (Figure 7B and Supplementary Table S3). The AA% was not significantly different, and the average values were 58.65 ± 11.91% for 1DC and 39.15 ± 15.7% for 2DC (Table 2 and Supplementary Table S3).

The C and N contents were significantly affected by an interaction between the time of day and zones of the algae (*p* < 0.05) in both daily cycles (Supplementary Figure S1, Supplementary Table S3). The N content had high values in 1DC at 13:00 h in the middle zone under attenuated and low radiation (Supplementary Figure S2A, Supplementary Table S3). In stark contrast, in 2DC, the N content peaked at 13:00 h in the canopy zone for all the treatments (Supplementary Figure S2B, Supplementary Table S3).

### Pearson Correlations

In both daily cycles, positive relationships between the photosynthetic and biochemical parameters were found. In 1DC, the correlations were positive between productivity and *in situ* electron transport rate (ETR _*in situ*_) (*r* = 0.99; *p* < 0.05). Chl*a* was positively correlated to *Fux* (*r*^2^ = 0.365; *p* < 0.05) and Chl_*c*1+*c*2_ (*r*^2^ = 0.341; *p* < 0.05) (Supplementary Table S4). In 2DC, Chl*a* was again positively correlated with Chl_*c*1+*c*2_ (*r*^2^ = 0.454; *p* < 0.05) and *Fux* (*r*^2^ = 0.426; *p* < 0.05), and similar patterns between Chl_*c*1+*c*2_ with *Fux* (*r*^2^ = 0.986; *p* < 0.05) were also detected. Lastly, a positive association between PC and AA% (*r*^2^ = 0.579; *p* < 0.05) was also found (Supplementary Table S5).

## Discussion

In this study, several physiological and biochemical responses in the giant kelp *M. pyrifera* were revealed to have photoacclimation patterns in response to changes solely in solar attenuation within the water column in a mesocosm system located in southern Chile. Specifically, *M. pyrifera* showed a vertical response within its thalli due to predictable variation in irradiance with depth. Indeed, PAR irradiance changes in the water column drive this species to behave as a highly light-adapted alga in the canopy zone and as a low light-adapted alga in the low exposure or down zone, as reported previously under natural conditions for different depths in the water column (Gómez et al., 2004; Gómez and Huovinen, 2011; Palacios et al., 2021). Here, we demonstrated that *M. pyrifera* responds differently to changes in light conditions through differential photoacclimation along its thalli. The results of photosynthetic capacity estimated as ETR in the thalli of *M. pyrifera* demonstrated sharp negative decreases with depth related solely to the diminishing light. Nevertheless, algal photosynthetic capacity estimated as *in situ* ETR was reduced by between 57 and 79% in the middle zone blades and from 79 to 93% in the bottom blades. A lesser reduction of 20% of the juvenile sporophytes has been found in another field study (Umanzor et al., 2020). This effect is only a consequence of light reaching the bottom of the tank because nutrients, temperature, pH, and salinity were the same at all depths in the experimental tanks.

Canopy zones of *M. pyrifera* exposed to high PAR showed photosynthetic differences in almost all parameters when compared with the middle and down zones. Canopy zones showed higher ETR _*in situ*_, indicating that these blades behave much like an alga adapted to strong sunlight. Middle zone blades behave as a transition between high and low irradiance. This different pattern can be related to different daily integrated irradiance (DIE) in the two daily cycles (Figures 2–4), i.e., in DC1, under total radiation, the ratio of DIE between the canopy and the down zone was 3.326, whereas, in DC2, was about two times higher (6.47) (Figure 2).

Regarding *M. pyrifera*, the canopy of this alga responded as a sunshine-adapted alga, whereas its subcanopy portion behaved as a shade-adapted alga (Colombo-Pallotta et al., 2006; Varela et al., 2018). Accordingly, Celis-Plá et al. (2014) described a higher ETR_max_ in *Cystoseira tamariscifolia* among algae collected from rocky shores with sun exposure than those collected in rocky pools with shaded solar exposure in two seasonal periods (winter and summer). Our *in situ* measurements revealed that *M. pyrifera* canopies are exposed throughout the daily cycle to irradiances higher than 850-?mol photons m^−2^ s^−1^. This value exceeded the saturated irradiance (Ek) measured in the laboratory through the RLC, which ranged from 200 to 300-μmol photons m^−2^ s^−1^ in both daily cycles at noon (data not shown).

Moving deeper in the water column, photosynthetic capacity starts to decrease toward the subcanopy due to a significant reduction in the irradiance driven by the Kd. The ETR_*in situ*_ decline is explained by light attenuation in the water column. Varela et al. (2018) reported a similar productivity pattern in *M. pyrifera*, where those algae cultivated in shallower waters had higher productivity than those cultivated at 6-m depths, a disparity they attributed to reduced irradiance. Similarly, in other work where productivity was also measured in the canopy/exposure zones and sub-canopy/low exposure of *M. pyrifera*, Colombo-Pallotta et al. (2006) uncovered a gradient of productivity, in that the high-exposure frond was the most productive part of the thalli while productivity was lower in the subcanopy portion. Plant productivity in μmolCm^−2^s^−1^ derived from the ETR _*in situ*_ was higher than the ETR_max_ (Jerez et al., 2016). Marambio et al. (2017) show the increase of the rETR_max_ in the apical fronds in spring, autumn, and winter. Similarly, ETR_max_ values in *Ulva rigida* were also higher when determining *Y*_*II*_ under solar radiation than under artificial actinic light (Longstaff et al., 2002; Figueroa et al., 2021). A possible explanation is that, under solar radiation, not only chlorophyll but also accessory pigments can be excited, which transfers photons to chlorophyll to a greater extent than under blue actinic artificial light. So, it is possible that one or more pieces of alga may present a more pronounced reduction in effective yield as a function of irradiance due to the cumulative dose in the measuring chamber.

*In situ*, we found that the photosynthetic capacity of *M. pyrifera* was about 75% higher in its canopy than its subcanopy portion in the deeper part of the tanks. Thus, the ETR in a high-exposure frond is 3-fold higher than a low-exposure frond due to less irradiance. There is a corresponding gradient in alga productivity through the water column from top to bottom that is causally related to diminishing irradiance and not to temperature or nutrient availability. Bordeyne et al. (2017) showed that the productivity in brown algae can function differently during the tide cycle, and the authors provide relevant information about immersion and emersion. These authors showed that, in these periods, primary production and respiration varied seasonally, with minimum values in winter and maximum values in summer, and these values were 5 and 3.5 times higher, respectively, when the community was exposed to air than when immersed. The floating kelp canopy of *M. pyrifera* is partly exposed to air, moved by the wind and waves, and the resultant effects on the photosynthetic rate are not well-understood under these more realistic conditions. In our case, the canopy was exposed to the air conditions, and the bubbling as the wave action in nature prevents that desiccation effect of the floating blades. The shifting gradients of nutrients and temperature could also influence the productivity of plants, both aquatic and terrestrial. We know of the canopy in the emersion time, maybe, can exhibit a lower photosynthetic activity (Bordeyne et al., 2015); in the same context, Golléty et al. (2008) suggest that the primary production and respiration can be related to the environmental factors as light and temperature seasonal variations, without neglecting the immersion periods when, indeed, some of them exhibiting a major part of their production when emersed (Quadir et al., 1979). In our experiment, however, the effect of light was separated from any nutrient and temperature effects with natural trends, since the mesocosm water columns were homogenized by air, ensuring that there were no other differences between tanks and treatments during the experiment.

The highest pigment contents were found in the canopy zone during the morning of the 1DC in the full radiation and attenuated treatments, whereas chlorophyll *a* tends to homogenize in the middle and down zones of the thalli. In 2DC, the pigment contents showed the same values throughout the thalli. In contrast, Varela et al. (2018) found differences with respect to the concentrations of the pigments (Chl*a*, Chl*c*, and *Fux*) in winter between different sporophytes cultivated at different water depths. The photoprotection pattern of phenolic compounds in *M. pyrifera* indicated the adaptation to sunlight by the alga, given the differences uncovered along its thalli. Work by Celis-Plá et al. (2014, 2015, 2016) in other brown algae revealed a higher presence of phenolic compounds during winter compared with summer, mainly due to the necessity of photoprotection against higher irradiance. Accordingly, we suspect this may explain the different responses of these compounds in *M. pyrifera* with respect to their positioning in the water column.

Despite certain differences in polyphenol content, no significant differences in antioxidant capacity during the experiments were detected. Rather, antioxidant capacity remained at 50% in both high- and low-exposure fronds in the first daily cycle, whereas, in the second cycle, this was somewhat lower, at about 30%, but not significantly different from the first daily cycle. This finding contrasts with positive correlations between phenolic compounds and antioxidant activity found in many other studies (Abdala-Díaz et al., 2014; Figueroa et al., 2014; Celis-Plá et al., 2016; Gómez et al., 2016; Beratto-Ramos et al., 2019, Zúñiga et al., 2021). The low antioxidant activity in the second daily cycle was also related to a decreased ETR_*in situ*_. Thus, antioxidant activity seems to be linked somehow to photosynthetic activity, and this has been reported in a few other habitat forming species of macroalgae (e.g., Celis-Plá et al., 2014, 2016; Zúñiga et al., 2021). Palacios et al. (2021) showed that the *M. pyrifera* grows in channels and fjords of the Chilean Patagonia, where they are adapted to shade due to a sharp gradient of turbidity and light availability. However, the authors did not find intra-thallus variation in biochemical content along the vertical profile could be found, except that the algae growing at 6-m depth resembled those at 13-m depth (Palacios et al., 2021).

## Conclusions

During the winter time, *M. pyrifera* shows a strong capacity to adapt to changing conditions in the water column of a mesocosm with different light gradients. This plastic ability to engage in various physiological strategies to cope with changing light conditions indicates that *M. pyrifera* can use resources present in the water column at different rates, enabling it to live in different habitats and variable environments, a prime example being places with differing levels of light penetration. *M. pyrifera* presents different strategies within the same sporophyte that fosters photo-acclimation or changes in photosynthetic performance according to differing solar irradiance conditions. This capacity can explain the high photosynthetic activity and photoprotection of the alga, which enable it to achieve high productivity and efficiency (Buschmann et al., 2014a). Mesocosm experiments have proved to be robust and insightful tools for better understanding the specific ecophysiological responses to environmental stressors of large and morphologically complex marine algae.

## Data Availability Statement

The original contributions presented in the study are included in the article/Supplementary Materials, further inquiries can be directed to the corresponding author/s.

## Author Contributions

AB, PC-P, and FF: investigation, conceptualization, formal analysis, writing the original draft, and subsequently editing it. JK, SP, and MH-G: experimental research, conceptualization, formal analysis, statistical analysis, writing, and editing the original draft. KV and RA: experimental research. All authors contributed to the article and approved the submitted version.

## Conflict of Interest

The authors declare that the research was conducted in the absence of any commercial or financial relationships that could be construed as a potential conflict of interest.
