# Supplementary material for: Solar Radiation as an Isolated Environmental Factor in an Experimental Mesocosm Approach for Studying Photosynthetic Acclimation of Macrocystis pyrifera (Ochrophyta)
Source: Front Plant Sci. 2021 Jul 2;12:622150. doi: 10.3389/fpls.2021.622150 (PMC8283697; doi:10.3389/fpls.2021.622150)
Supplement: Supplementary file 1 [file Data_Sheet_1.docx]

**SUPPLEMENTARY MATERIAL**

**Table S1** Water temperature in the mesocosms system, in the first and second daily cycle, in relation to total, attenuated, and low radiation treatments, and air temperature in the surroundings of the mesocosm.

|  | **time** | **total** | **attenuated** | **low** | **Air** |
| --- | --- | --- | --- | --- | --- |
| DC. 1 | 08:00 a.m. | 8.57 ± 0.01 | 8.50 ± 0.01 | 8.81 ± 0.02 | 8.83 ± 0.16 |
|  | 13:00 p.m. | 13.22 ± 0.32 | 12.59 ± 0.23 | 12.44 ± 0.23 | 17.53 ± 0.3 |
|  | 17:00 p.m. | 13.45 ± 0.1 | 12.83 ± 0.06 | 13.15 ± 0.07 | 12.56 ± 0.28 |
| DC. 2 | 08:00 am | 5.64 ± 0.05 | 5.66 ± 0.08 | 6.21 ± 0.04 | 1.90 ± 0.11 |
|  | 13:00 p.m. | 10.70 ± 0.32 | 10.08 ± 0.23 | 10.29 ± 0.24 | 15.61 ± 1.34 |
|  | 17:00 p.m. | 10.94 ± 0.13 | 10.31 ± 0.11 | 10.88 ± 0.11 | 8.84 ± 0.31 |

**Table S2** nitrate concentration (µM) in the mesocosms system along to the water column, in wintertime.

| **water column** | **Average** | **SE** |
| --- | --- | --- |
| high | 4.693293 | 0.082575 |
| middle | 5.133974 | 0.231716 |
| low | 4.189417 | 0.212336 |

**Table S3** ANOVA results after experimental period testing for the effect of Treatments, time, and Part of thalli of the phenolic compounds, antioxidant activity, chlorophyll *a*, chlorophyll *c1+c2*, fucoxanthin, ratio carbon: nitrogen, carbon, y nitrogen of *Macrocystis pyrifera* in two dayle cycles. Significant differences at α < 0.05 are shown in asterisk.

| ***Macrocystis pyrifera*** | | | | | | | | | | |
| --- | --- | --- | --- | --- | --- | --- | --- | --- | --- | --- |
|  |  | ***DC 1*** | | | | ***DC 2*** | | | | |
|  |  | *df* | *MS* | *F* | *P* |  | *df* | *MS* | *F* | *P* |
| ***Phenolics*** | *Treatments (T)* | 2 | 165.02 | 15.99 | ***** |  | 2 | 142.43 | 6.15 | ***** |
| ***compounds*** | *time (t)* | 2 | 25.60 | 2.48 | ***** |  | 2 | 77.55 | 3.35 | ***** |
|  | *Part of thalli (Pt)* | 2 | 146.14 | 14.16 | ***** |  | 2 | 59.84 | 2.59 | ***** |
|  | *T*t* | 4 | 29.77 | 2.88 | ***** |  | 4 | 20.07 | 0.87 |  |
|  | *T*Pt* | 4 | 10.21 | 0.99 |  |  | 4 | 12.69 | 0.55 |  |
|  | *t*Pt* | 4 | 1.36 | 0.13 |  |  | 4 | 29.66 | 1.28 |  |
|  | *T*t*Pt* | 8 | 23.59 | 2.29 | ***** |  | 8 | 11.62 | 0.50 |  |
|  | *Res* | 81 | 10.32 |  |  |  | 81 | 23.14 |  |  |
| ***AA%*** | *Treatments (T)* | 2 | 781.80 | 5.71 | ***** |  | 2 | 2212.07 | 9.26 | ***** |
|  | *time (t)* | 2 | 35.64 | 0.26 |  |  | 2 | 118.1623 | 0.49 |  |
|  | *Part of thalli (Pt)* | 2 | 123.93 | 0.91 |  |  | 2 | 404.9011 | 1.69 |  |
|  | *T*t* | 4 | 119.95 | 0.88 |  |  | 4 | 60.62978 | 0.25 |  |
|  | *T*Pt* | 4 | 131.47 | 0.96 |  |  | 4 | 202.3236 | 0.85 |  |
|  | *t*Pt* | 4 | 64.28 | 0.47 |  |  | 4 | 64.15835 | 0.27 |  |
|  | *T*t*Pt* | 8 | 120.13 | 0.88 |  |  | 8 | 30.79988 | 0.13 |  |
|  | *Res* | 81 | 136.80 |  |  |  | 81 | 239.0043 |  |  |
| ***Chla*** | *Treatments (T)* | 2 | 0.04 | 2.15 |  |  | 2 | 0.01 | 0.02 |  |
|  | *time (t)* | 2 | 0.10 | 5.70 | ***** |  | 2 | 287.54 | 578.26 | ***** |
|  | *Part of thalli (Pt)* | 2 | 0.20 | 11.19 | ***** |  | 2 | 572.27 | 1150.85 | ***** |
|  | *T*t* | 4 | 0.03 | 1.88 |  |  | 4 | 11.91 | 23.94 | ***** |
|  | *T*Pt* | 4 | 0.03 | 1.58 |  |  | 4 | 0.36 | 0.72 |  |
|  | *t*Pt* | 4 | 0.07 | 4.00 | ***** |  | 4 | 186.32 | 374.70 | ***** |
|  | *T*t*Pt* | 8 | 0.06 | 3.38 | ***** |  | 8 | 9.97 | 20.05 | ***** |
|  | *Res* | 81 | 0.02 |  |  |  | 81 | 0.50 |  |  |
| ***Chl_c1+c2_*** | *Treatments (T)* | 2 | 0.06 | 0.57 |  |  | 2 | 0.01 | 0.15 |  |
|  | *time (t)* | 2 | 0.19 | 1.66 |  |  | 2 | 0.02 | 0.46 |  |
|  | *Part of thalli (Pt)* | 2 | 0.03 | 0.26 |  |  | 2 | 0.01 | 0.27 |  |
|  | *T*t* | 4 | 0.09 | 0.76 |  |  | 4 | 0.01 | 0.31 |  |
|  | *T*Pt* | 4 | 0.01 | 0.08 |  |  | 4 | 0.01 | 0.23 |  |
|  | *t*Pt* | 4 | 0.02 | 0.22 |  |  | 4 | 0.00 | 0.04 |  |
|  | *T*t*Pt* | 8 | 0.04 | 0.32 |  |  | 8 | 0.01 | 0.15 |  |
|  | *Res* | 81 | 0.11 |  |  |  | 81 | 0.04 |  |  |
| ***Fuco*** | *Treatments (T)* | 2 | 0.01 | 0.25 |  |  | 2 | 0.00 | 0.27 |  |
|  | *time (t)* | 2 | 0.03 | 1.11 |  |  | 2 | 0.00 | 0.44 |  |
|  | *Part of thalli (Pt)* | 2 | 0.01 | 0.27 |  |  | 2 | 0.00 | 0.33 |  |
|  | *T*t* | 4 | 0.00 | 0.07 |  |  | 4 | 0.00 | 0.30 |  |
|  | *T*Pt* | 4 | 0.00 | 0.16 |  |  | 4 | 0.00 | 0.24 |  |
|  | *t*Pt* | 4 | 0.00 | 0.12 |  |  | 4 | 0.00 | 0.06 |  |
|  | *T*t*Pt* | 8 | 0.00 | 0.08 |  |  | 8 | 0.00 | 0.15 |  |
|  | *Res* | 81 | 0.03 |  |  |  | 81 | 0.01 |  |  |
| ***C:N*** | *Treatments (T)* | 2 | 1.57 | 2.29 |  |  | 2 | 19.40 | 17.59 | ***** |
|  | *time (t)* | 2 | 0.20 | 0.30 |  |  | 2 | 3.27 | 2.96 | ***** |
|  | *Part of thalli (Pt)* | 2 | 11.89 | 17.41 | ***** |  | 2 | 6.43 | 5.83 | ***** |
|  | *T*t* | 4 | 0.30 | 0.44 |  |  | 4 | 1.40 | 1.27 |  |
|  | *T*Pt* | 4 | 0.65 | 0.95 |  |  | 4 | 1.54 | 1.39 |  |
|  | *t*Pt* | 4 | 2.28 | 3.34 | ***** |  | 4 | 1.63 | 1.48 |  |
|  | *T*t*Pt* | 8 | 1.77 | 2.60 |  |  | 8 | 1.65 | 1.49 |  |
|  | *Res* | 54 | 0.68 |  |  |  | 54 | 1.10 |  |  |
| ***C*** | *Treatments (T)* |  |  |  |  |  | 2 | 570.71 | 7.27 | ***** |
|  | *time (t)* | 2 | 24.91 | 0.51 |  |  | 2 | 886.63 | 11.30 | ***** |
|  | *Part of thalli (Pt)* | 2 | 25.10 | 0.52 |  |  | 2 | 351.64 | 4.48 | ***** |
|  | *T*t* | 4 | 17.86 | 0.37 |  |  | 4 | 44.96 | 0.57 |  |
|  | *T*Pt* | 4 | 325.55 | 6.69 | ***** |  | 4 | 179.69 | 2.29 |  |
|  | *t*Pt* | 4 | 345.99 | 7.11 | ***** |  | 4 | 272.55 | 3.47 | ***** |
|  | *T*t*Pt* | 8 | 64.97 | 1.34 | * |  | 8 | 22.10 | 0.28 | * |
|  | *Res* | 54 | 48.64 |  |  |  | 54 | 78.49 |  |  |
| ***N*** | *Treatments (T)* | 2 | 8.27 | 3.13 |  |  | 2 | 34.55 | 10.95 | ***** |
|  | *time (t)* | 2 | 0.54 | 0.20 |  |  | 2 | 0.41 | 0.13 |  |
|  | *Part of thalli (Pt)* | 2 | 42.21 | 15.95 | ***** |  | 2 | 24.38 | 7.72 | ***** |
|  | *T*t* | 4 | 1.15 | 0.43 |  |  | 4 | 3.63 | 1.15 |  |
|  | *T*Pt* | 4 | 2.55 | 0.96 |  |  | 4 | 4.28 | 1.36 |  |
|  | *t*Pt* | 4 | 10.93 | 4.13 | ***** |  | 4 | 8.66 | 2.74 | ***** |
|  | *T*t*Pt* | 8 | 7.19 | 2.72 | ***** |  | 8 | 3.57 | 1.13 | * |
|  | *Res* | 54 | 2.65 |  |  |  | 54 | 3.16 |  |  |
| *ETRin situ* | *Treatments (T)* | 2 | 15160.1 | 25.7 | ***** |  | 2 | 9310.3 | 63.2 | ***** |
|  | *time (t)* | 2 | 1804521.8 | 3060.8 | ***** |  | 2 | 1007803.2 | 6836.8 | ***** |
|  | *Part of thalli (Pt)* | 2 | 624644.8 | 1059.5 | ***** |  | 2 | 423648.8 | 2874.0 | ***** |
|  | *T*t* | 4 | 10947.5 | 18.6 | ***** |  | 4 | 5588.5 | 37.9 | ***** |
|  | *T*Pt* | 4 | 10188.6 | 17.3 | ***** |  | 4 | 4056.2 | 27.5 | ***** |
|  | *t*Pt* | 4 | 454068.6 | 770.2 | ***** |  | 4 | 266821.6 | 1810.1 | ***** |
|  | *T*t*Pt* | 8 | 8515.8 | 14.4 | ***** |  | 8 | 2799.6 | 19.0 | ***** |
|  | *Res* | 945 | 589.6 |  |  |  | 945 | 147.4 |  |  |
| *Res: Residual* | |  |  |  |  |  |  |  |  |  |

**Table S4** Pearson correlation of the first daily cycle (average of the treatments), respect to the PC, Chla, Chlc1+c2, Fux, AA%, ETR*_in situ_*, C and N.

|  | ***PC*** | ***Chla*** | ***Chl _c1+c2_*** | ***Fuc*** | ***AA%*** | ***ETR_in situ_*** | ***C*** | ***N*** |
| --- | --- | --- | --- | --- | --- | --- | --- | --- |
| ***PC*** |  | -0.267 | 0.00575 | 0.0249 | 0.534 | -0.161 | 0.039 | -0.219 |
|  |  | 0.0161 | 0.959 | 0.825 | 2.8E-07 | 0.15 | 0.729 | 0.05 |
| ***Chla*** |  |  | 0.341 | 0.304 | -0.314 | 0.0503 | 0.0367 | 0.145 |
|  |  |  | 0.00183 | 0.0058 | 0.00433 | 0.656 | 0.745 | 0.196 |
| ***Chl _c1+c2_*** |  |  |  | 0.998 | -0.197 | -0.00101 | -0.223 | -0.0323 |
|  |  |  |  |  | 0.0774 | 0.993 | 0.0451 | 0.775 |
| ***Fuc*** |  |  |  | -0.171 | -0.0134 | -0.223 | -0.0356 |  |
|  |  |  |  |  | 0.127 | 0.906 | 0.0456 | 0.752 |
| ***AA%*** |  |  |  |  |  | -0.0968 | 0.0885 | -0.105 |
|  |  |  |  |  |  | 0.39 | 0.432 | 0.35 |
| ***ETR_in situ_*** |  |  |  |  |  |  | -0.0392 | -0.114 |
|  |  |  |  |  |  |  | 0.728 | 0.309 |
| ***C*** |  |  |  |  |  |  |  | 0.397 |
|  |  |  |  |  |  |  |  | 0.000244 |

**Table S5** Pearson correlation of the second daily cycle (average of the treatments), respect to the PC, Chla, Chl*_c1+c2_*, Fuc, AA%, ETR*_in situ_*, C and N.

|  | ***PC*** | ***Chla*** | ***Chl_c1+c2_*** | ***Fuc*** | ***AA%*** | ***ETR_in situ_*** | ***C*** | ***N*** |
| --- | --- | --- | --- | --- | --- | --- | --- | --- |
| ***PC*** |  | 0.0262 | -0.0633 | -0.0829 | 0.579 | -0.198 | -0.0371 | -0.0433 |
|  |  | 0.816 | 0.575 | 0.462 | 1.47E-08 | 0.0768 | 0.742 | 0.701 |
| ***Chla*** |  |  | 0.454 | 0.426 | -0.168 | -0.0717 | -0.0135 | 0.208 |
|  |  |  | 0.0000205 | 0.0000737 | 0.135 | 0.525 | 0.905 | 0.0627 |
| ***Chl_c1+c2_*** |  |  |  | 0.958 | -0.118 | 0.00967 | 0.0524 | 0.144 |
|  |  |  |  | 1.29E-41 | 0.295 | 0.932 | 0.642 | 0.2 |
| ***Fuc*** |  |  |  |  | -0.0895 | 0.0178 | 0.0428 | 0.114 |
|  |  |  |  |  | 0.427 | 0.875 | 0.705 | 0.309 |
| ***AA%*** |  |  |  |  |  | -0.0858 | 0.00124 | -0.0795 |
|  |  |  |  |  |  | 0.446 | 0.991 | 0.481 |
| ***ETR_in situ_*** |  |  |  |  |  |  | 0.153 | -0.0732 |
|  |  |  |  |  |  |  | 0.171 | 0.516 |
| ***C*** |  |  |  |  |  |  |  | 0.288 |
|  |  |  |  |  |  |  |  | 0.00923 |

**Figure S1** Internal carbon content in *Macrocystis pyrifera* fronds in the (A) first daily cycle and (B) second daily cycle experiment under different solar irradiance treatments: total, attenuated, and low radiation at 8:00, 13:00, and 17:00 h. The C was determined in three zones of the alga: canopy, middle, and down (mean ± SE, n = 3). Lower-case letters denote significant differences after the SNK test.


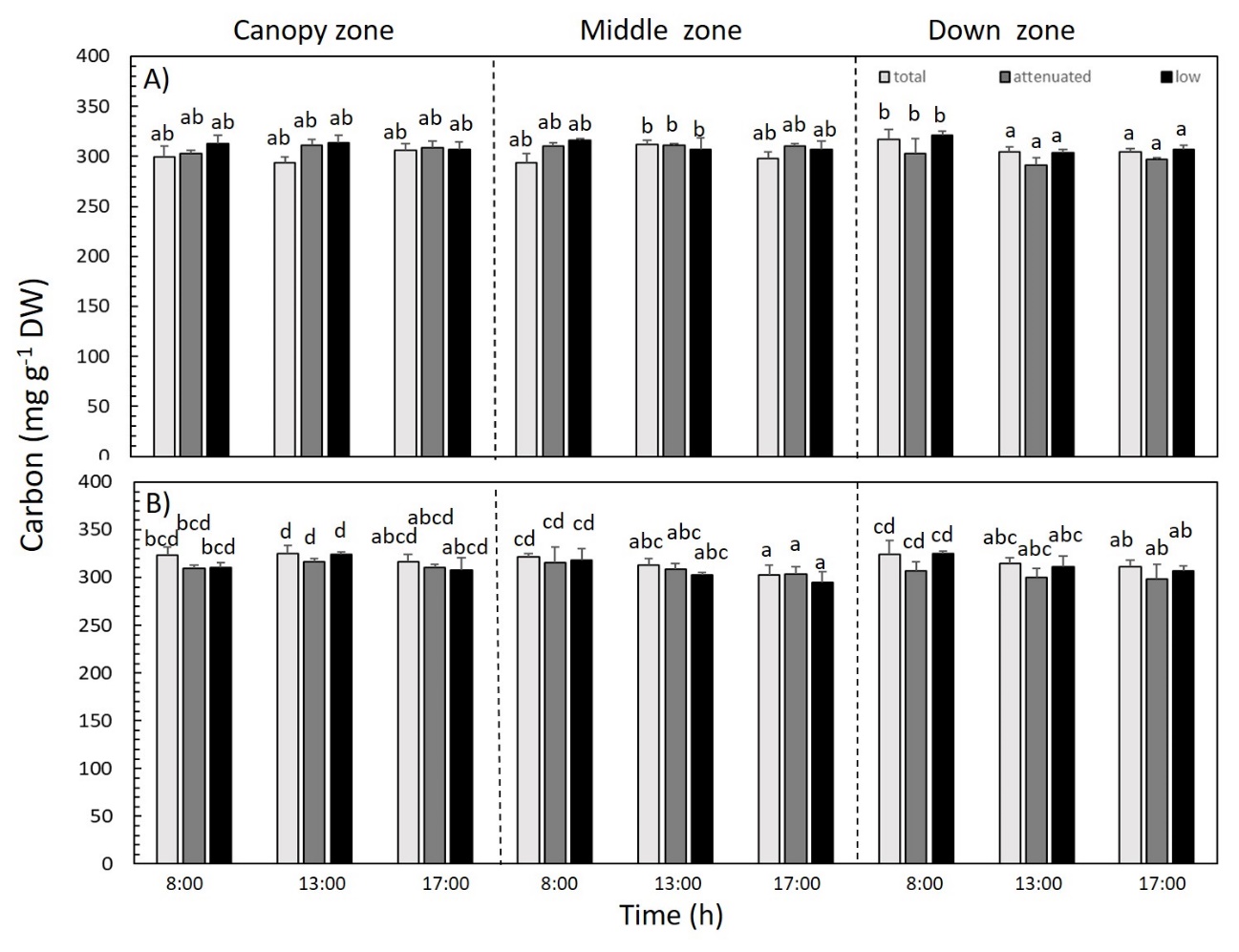


**Figure S2** Internal nitrogen content in *Macrocystis pyrifera* fronds in the (A) first daily cycle and (B) second daily cycle experiment under different solar irradiance treatments: total, attenuated, and low radiation at 8:00, 13:00, and 17:00 h. The N was determined in three zones of the alga: canopy, middle, and down (mean ± SE, n = 3). Lower-case letters denote significant differences after the SNK test.

**
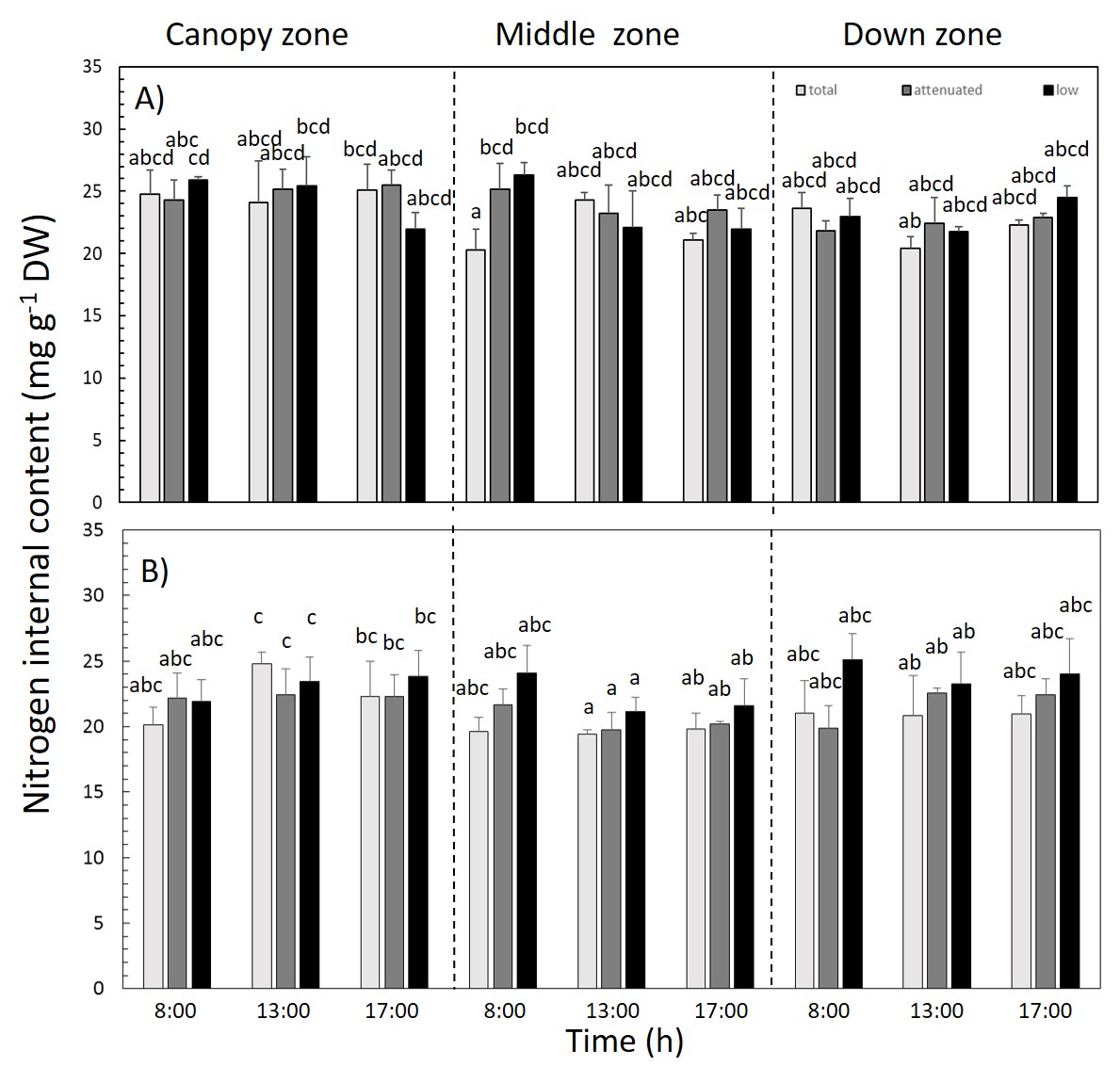
**
